# Management of an Immature Necrotic Permanent Molar with Apical Periodontitis Treated by Regenerative Endodontic Protocol Using Calcium Hydroxide and MM-MTA: A Case Report with Two Years Follow Up

**DOI:** 10.3390/dj7010001

**Published:** 2019-01-01

**Authors:** Jessy Ajram, Issam Khalil, Richard Gergi, Carla Zogheib

**Affiliations:** 1 Faculty of Dentistry, Department of Endodontics, Saint Joseph University, Beirut 1107-2180, Lebanon; Issamtkhalil@gmail.com (I.K.); drrichardgergi@hotmail.com (R.G.); 2 Head of Department of Endodontics, Saint Joseph University, Beirut 1107-2180, Lebanon; Zogheibcarla@gmail.com

**Keywords:** calcium hydroxide, Cone Beam Computed Tomography, immature necrotic permanent tooth, MM-MTA, regenerative endodontic treatment

## Abstract

Traditionally, immature teeth diagnosed with necrotic pulp and periapical periodontitis were treated by apexification with long-term calcium hydroxide or in one session with mineral trioxide aggregate (MTA) or Biodentine apical plug. However, these teeth become fragile and susceptible to root fracture. Regenerative endodontic procedure is a new therapeutic approach that promotes continuation of root growth in immature necrotic teeth potentially preventing root fracture. Only few case reports have shown the success of this procedure on molar cases. The current case report demonstrates a regeneration of a lower first molar with necrotic pulp and chronic apical abscess treated with Micro Mega-MTA (MM-MTA), a new endodontic biomaterial that has not been described previously. Calcium hydroxide was used as an intracanal medicament for two weeks. Next, calcium hydroxide was removed and after blood clot creation, MM-MTA^®^ was placed over it. Apical healing and continuation of root growth were evident at nine months follow-up. CBCT at two years follow-up confirmed apical closure and complete healing. This case shows that a regenerative endodontic procedure for management of an immature necrotic permanent molar is feasible and can be successfully done using Ca(OH)_2_ and MM-MTA.

## 1. Introduction

Treatment of immature necrotic teeth is clinically challenging. Treatment options include apexification procedures using of calcium hydroxide (Ca(OH)_2_) to induce formation of a calcific barrier at the apex or placement of mineral trioxide aggregate plug (MTA) [1,2,3,4]. Both techniques achieve clinical success but do not allow further root development, leaving the tooth susceptible to fracture [4,5,6].

More recently, regenerative endodontic treatment (RET) presented as an alternative treatment option that allows continued root formation and apical closure, following a successful disinfection of the root canal [7,8,9,10,11,12,13]. Regenerative endodontics is defined as “restoration of tissue architecture and biological function of damaged tissues by tissue similar to the original tissue” [14]. This treatment usually begins with chemical disinfection of the canals. Abundant irrigation with ethylene diamine tetra-acidic acid (EDTA) is the initial step. EDTA allows the release of the growth factors from dentin. The irrigation is followed by root canal dressing with triple antibiotic paste (TAP) or calcium hydroxide (Ca(OH)_2_). Hemorrhage is then induced into the root canal to form a blood clot that provides growth factors for the cells and acts as a scaffold [8,15]. The last step consists of sealing the canal orifice with MTA or Biodentine, which allows the regeneration of new tissue adjacent to it. Finally, the permanent coronal restoration is placed.

Ca(OH)_2_ has been successfully used for root canal disinfection before regenerative endodontic procedures [16,17,18]. However, reports with Ca(OH)_2_ medication are limited, particularly in molar teeth [19]. The use of MM-MTA a new endodontic biomaterial instead of MTA in molar teeth has not been previously reported.

This case report describes a successful regenerative endodontic therapy performed on an immature permanent molar with necrotic pulp and symptomatic apical periodontitis, using Ca(OH)_2_ as an intracanal medication and MM-MTA^®^ (Micro Mega, Besançon, CEDEX, France) for coronal sealing [20].

## 2. Case Presentation

Based on the institution’s guidelines, approval from the ethics committee is not required for individual case description. A written consent has been obtained from the patient’s mother authorizing the use of the medical information and imaging.

**Figure 1:**
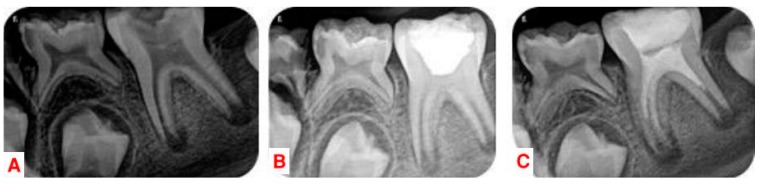
(**A**) Preoperative radiograph. (**B**) Placement of Ca(OH)_2_ in the pulp chamber. (**C**) Postoperative radiograph after root canal disinfection, bleeding induction, placement of MM-MTA over blood clot, placement of coronal restoration.

A healthy 7-year-old girl presented with a history of swelling and pain of the left side of the mandibular region. Clinical examination showed dentin caries lesion on the mandibular left first molar with notable localized swelling in the buccal mucosa. The cold test for the affected molar was negative. The molar was extremely sensitive to percussion and palpation with class I tooth mobility. Radiographic examination showed radiolucent periapical lesions adjacent to the distal and mesial roots with immature wide open apices (Figure 1). A common diagnosis of pulp necrosis with symptomatic apical periodontitis was made. After explaining all treatment options (apexification with MTA, RET), risks and benefits, an informed consent was obtained from the patient’s parents and the decision was to attempt RET with both Ca(OH)_2_ and MM-MTA.

### 2.1. First Appointment

The patient was very cooperative. The pulp chamber was accessed after local anesthetic injection with 3% mepivacaine (Septodont, CEDEX, Saint-Maur-des-fosses, France) without vasoconstrictor and rubber dam isolation. Each root canal orifice was gently irrigated with 10 mL of 2.5% sodium hypochlorite (NaOCl) without any instrumentation followed by 5 mL of 20% EDTA (Pulpdent, Watertown, MA, USA) activated with EndoActivator (Dentsply Tulsa Dental, Tulsa, OK, USA) for 1 min in the coronal third. NaOCl and EDTA were delivered via a 27-gauge closed ends and side vents positioned approximately 1–2 mm below the root canal orifice. Ca(OH)_2_ powder (Merck, Darmstadt, Germany) was mixed with sterile water in a 3:1 ratio to produce a thick, homogeneous paste. The mixture was placed in the pulp chamber and was loosely packed into the coronal portion of the root canals with moist cotton pellets (Figure 1). The access cavity was sealed with Cavit (3M Espe, St. Paul, MN, USA).

### 2.2. Second Appointment

After 3 weeks, the patient came for treatment completion. Clinical examination revealed no pain to percussion/palpation and no soft-tissue swelling. The tooth was similarly anesthetized with 3% mepivacaine (Septodont, CEDEX, Saint-Maur-des-fosses, France) without vasoconstrictor to facilitate bleeding, and isolated with a rubber dam, and reaccessed. Ca(OH)_2_ paste was removed with copious distilled water. Root canals were irrigated with 5 mL of 20% EDTA for 5 min followed by a 10 mL sterile saline. Apical bleeding was induced by gentle irritation with #15 K-files (Dentsply Maillefer, Ballaigues, Switzerland). The file was placed a few millimeters beyond the apical foramen and bleeding was induced up to 3 mm from the cemento-enamel junction (CEJ). After formation of the blood clot, MM-MTA (Micro-Mega, Besançon CEDEX, France) was prepared according to the manufacturer’s instructions and was gently adapted over the blood clot. The tooth was sealed with glass ionomer cement (Glasionomer FX-II; Shofu Inc., Kyoto, Japan) to allow the MM-MTA to set. After twenty minutes, the glass ionomer was removed and the access cavity was restored using resin composite (Z350; 3M ESPE, St. Paul, MN, USA) (Figure 1).

### 2.3. Follow Up

**Figure 2:**
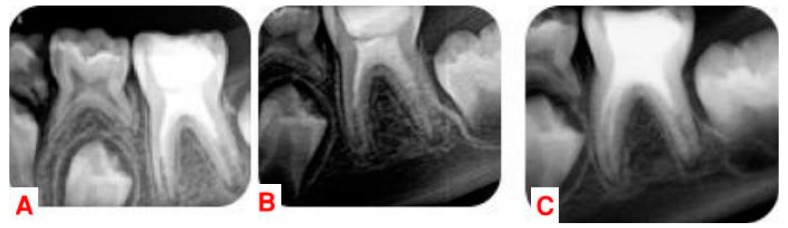
(**A**) Recall radiograph at three months. (**B**) Recall radiograph at nine months. (**C**) Recall radiograph at twelve months.

**Figure 3:**
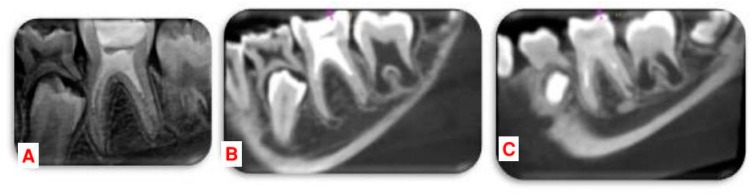
Radiographs at two years (**A**) Recall 2D radiograph at two years. (**B**,**C**) cone-beam computed tomography (CBCT) images in oblique view of the mesial and distal roots.

In the 3, 9, 12 and 24-month follow-ups, the tooth was asymptomatic. Clinical examination showed physiological mobility and absence of sensitivity to percussion and palpation. The follow-up radiographs revealed complete periapical healing in the mesial and distal roots at 9 months, with apical closure as well as significant increase in root length and dentin thickness at 12 months. Cone-beam computed tomography (CBCT) (Carestream, CS8100) with a small field of view (FOV) was used at 24 months to assess radiographic changes after regenerative endodontic procedures, allowing for axial and oblique views of the roots and bone (Figure 2 and Figure 3).

## 3. Discussion

This case highlights the potential of the regenerative endodontic technique for treatment of immature necrotic permanent molar using Ca(OH)_2_ and MM-MTA. Key factors for a successful outcome are careful case selection and the choice of medications for disinfection and sealing.

In young children and with a large apical diameter greater than 1mm radiographically, RET shows better results compared to apexification [21,22]. Therefore, RET was the procedure of choice. No instrumentation was performed in the present case. Instrumentation is contraindicated in regenerative endodontic procedure based on the 2016 guidelines by the American Association of Endodontists [23]. The root dentinal walls are so thin and susceptible to fractures by any instrumentation. In addition, the formation of a smear layer can occlude the dentinal walls and tubules [13].

Disinfection of the root canal system was accomplished with 10 mL NaOCl irrigation (2.5%). A concentration of 2.5% of NaOCl has shown to be effective on necrotic and organic tissues and has an antimicrobial effect. Higher concentrations of NaOCl are not recommended due to cytotoxicity on the stem cells of the apical papillae (SCAP) [24,25].

Ca(OH)_2_ has been used as the intra-canal medication to prevent infection and improve root length and wall thickness as shown by Bose et al. [18]. A study by Althumairy et al. [26] showed a higher survival rate for the SCAP when dentine was exposed to Ca(OH)_2_ compared to the lowest recommended concentration of TAP (0.1 mg/mL). Furthermore, in many of the case reports where the TAP was used, allergies and tooth discoloration have been reported.

The liberation of growth factors from the dentinal matrix is essential for the differentiation of the stem cells. EDTA is a chelating agent that demineralizes the dentin, which results in the liberation of these growth factors. In addition, the lower survival rate of SCAP due to the use of NaOCl can be reversed by using 20% EDTA [24,25].

In most RET case studies, MTA is used as the coronal sealant. The sealing properties and excellent biocompatibility of MTA make it the material of choice for clot protection [27,28,29,30]. MM-MTA^®^ is a novel product that has similar benefits and properties like MTA. MM-MTA^®^ is composed primarily of Portland cement and bismuth oxide such as other MTA products. MM-MTA^®^ contains calcium carbonate and a chloride accelerator. These additives result in a shorter setting time (20 min) when comparing it to other MTA products [31]. In terms of biocompatibility, MM-MTA^®^ proved to be similar to MTA: either showed no or very limited toxic effects in vitro and similar biocompatibility in vivo [32]. A recent study showed that the three inductive biomaterials MTA, Biodentine and MM-MTA^®^ did not exhibit a cytotoxic effect on the human bone marrow stem cells and all materials stimulate osteogenic differentiation of human bone marrow stem cells [33]. 

Radiographic outcome using 2D images is in accordance with the reports of Chueh et al. [9] showing resolution of apical radiolucency within 3–21 months (mean = 8 months) and ‘‘nearly normal’’ root development 10–29 months (mean = 16 months) after REP. The most accurate method for the assessment of root development is 3-dimensional (3D) imaging because the root development occurs in a 3D pattern. Data on 3D assessment of root development after RET in immature teeth are very limited. Although CBCT scanning may expose the patient to higher levels of radiation, it can be used in a small FOV while providing the additional radiographic information needed. In this case, no preoperative CBCT images were taken. CBCT images at 24 months showed complete periapical healing and closure of the apical foramen on both mesial canals and distolingual canal. The distobuccal canal presented a resolution of the apical lesion without complete apical closure.

## 4. Conclusions

This case shows that regenerative endodontic technique for management of an immature necrotic permanent molar is feasible and can be successfully done using Ca(OH)_2_ and MM-MTA. Future clinical trials comparing RET with Ca(OH)_2_ in molar teeth to the traditional apexification techniques are needed to understand better the effect of RET on periapical healing in molar teeth.

## References

[1] Andreasen J.O., Farik B., Munksgaard E.C. (2002). Long-term calcium hydroxide as a root canal dressing may increase risk of root fracture. Dent. Traumatol..

[2] Thibodeau B., Trope M. (2007). Pulp revascularization of a necrotic infected immature permanent tooth: Case report and review of the literature. Pediatr. Dent..

[3] Trope M. (2010). Treatment of the immature tooth with a non-vital pulp and apical periodontitis. Dent. Clin. N. Am..

[4] Rafter M. (2005). Apexification: A review. Dent. Traumatol..

[5] Simon S., Rilliard F., Berdal A., Machtou P. (2007). The use of mineral trioxide aggregate in one-visit apexification treatment: A prospective study. Int. Endod. J..

[6] Witherspoon D.E., Small J.C., Regan J.D., Nunn M. (2008). Retrospective analysis of open apex teeth obturated with mineral trioxide aggregate. J. Endod..

[7] Iwaya S.I., Ikawa M., Kubota M. (2001). Revascularization of an immature permanent tooth with apical periodontitis and sinus tract. Dent. Traumatol..

[8] Banchs F., Trope M. (2004). Revascularization of immature permanent teeth with apical periodontitis: New treatment protocol?. J. Endod..

[9] Chueh L.H., Huang G.T. (2006). Immature teeth with periradicular periodontitis or abscess undergoing apexogenesis: A paradigm shift. J. Endod..

[10] Jeeruphan T., Jantarat J., Yanpiset K., Suwannapan L., Khewsawai P., Hargreaves K.M. (2012). Mahidol study 1: Comparison of radiographic and survival outcomes of immature teeth treated with either regenerative endodontic or apexification methods—A retrospective study. J. Endod..

[11] Jung I.Y., Lee S.J., Hargreaves K.M. (2008). Biologically based treatment of immature permanent teeth with pulpal necrosis: A case series. J. Endod..

[12] Ding R.Y., Cheung G.S., Chen J., Yin X.Z., Wang Q.Q., Zhang C.F. (2009). Pulp revascularization of immature teeth with apical periodontitis: A clinical study. J. Endod..

[13] Cehreli Z.C., Isbitiren B., Sara S., Erbas G. (2011). Regenerative endodontic treatment (revascularization) of immature necrotic molars medicated with calcium hydroxide: A case series. J. Endod..

[14] Kim S.G., Malek M., Sigurdsson A., Lin L.M., Kahler B. (2018). Regenerative endodontics: A comprehensive review. Int. Endod. J..

[15] Thibodeau B., Teixeira F., Yamauchi M., Caplan D.J., Trope M. (2007). Pulp revascularization of immature dog teeth with apical periodontitis. J. Endod..

[16] Petrino J.A., Boda K.K., Shambarger S., Bowles W.R., McClanahan S.B. (2010). Challenges in regenerative endodontics: A case series. J. Endod..

[17] Torabinejad M., Turman M. (2011). Revitalization of tooth with necrotic pulp and open apex by using platelet-rich plasma: A case report. J. Endod..

[18] Bose R., Nummikoski P., Hargreaves K. (2009). A retrospective evaluation of radiographic outcomes in immature teeth with necrotic root canal systems treated with regenerative endodontic procedures. J. Endod..

[19] Chueh L.H., Ho Y.C., Kuo T.C., Lai W.H., Chen Y.H., Chiang C.P. (2009). Regenerative endodontic treatment for necrotic immature permanent teeth. J. Endod..

[20] Khalil I.T., Naaman A., Sarkis T. (2013). MM-MTA for direct pulp capping: A histologic comparison with ProRoot MTA in rat molars. J. Contemp. Dent. Pract..

[21] Estefan B.S., El Batouty K.M., Nagy M.M., Diogenes A. (2016). Influence of age and apical diameter on the success of endodontic regeneration proceudres. J. Endod..

[22] Chen X., Bao Z.F., Liu Y., Liu M., Jin X.Q., Xu X.B. (2013). Regenerative endodontic treatment of an immature permanent tooth at an early stage of root development: A case report. J. Endod..

[23] American Association of Endodontists AAE Clinical Considerations for Regenerative Procedure. https://www.aae.org/specialty/clinical-resources/regenerative-endodontics/.

[24] Martin D.E., De Almeida J.F., Henry M.A., Khaing Z.Z., Schmidt C.E., Teixeira F.B., Diogenes A. (2014). Concentration-dependent effect of sodium hypochloride on stem cells of apical papilla survival and differentiation. J. Endod..

[25] Trevino E.G., Patwardhan A.N., Henry M.A., Perry G., Dybdal-Hargreaves N., Hargreaves K.M., Diogenes A. (2011). Effect of irrigants on the survival of human stem cells of the apical papilla in a platelet-rich plasma scaffold in human root tips. J. Endod..

[26] Althumairy R.I., Teixeira F.B., Diogenes A. (2014). Effect of dentin conditioning with intracanal medicaments on survival of stem cells of apical papilla. J. Endod..

[27] Maroto M., Barberia E., Planells P., Vera V. (2003). Treatment of a non vital immature incisor with mineral trioxide aggregate (MTA). Dent. Traumatol..

[28] Okiji T., Yoshiba K. (2009). Reparative dentinogenesis induced by mineral trioxide aggregate: A review from the biological and physicochemical points of view. Int. J. Dent..

[29] Camilleri J., Pitt Ford T. (2006). Mineral trioxide aggregate: A review of the constituents and biological properties of the material. Int. Endod. J..

[30] Maturo P., Costacurta M., Bartolino M., Docimo R. (2009). MTA applications in pediatric dentistry. Oral Implantol..

[31] Khalil I., Naaman A., Camilleri J. (2015). Investigation of a novel mechanically mixed Mineral Trioxide Aggregate (MM-MTA^TM^). Int. Endod. J..

[32] Khalil I., Isaac J., Chaccar C., Sautier J.M., Berdal A., Naaman N., Naaman A. (2012). Biocompatibility assessment of modified portland cement in comparison with MTA^®^: In vivo and in vitro studies. Saudi Endod. J..

[33] Margunato S., Tasli P.N., Aydin S., Karapınar Kazandağ M., Şahin F. (2015). In vitro evaluation of ProRoot MTA, Biodentine, and MM-MTA on human alveolar bone marrow stem cells in terms of biocompatibility and mineralization. J. Endod..

